# Birth-Rate Problem

**Published:** 1920-02-14

**Authors:** 


### Birth-rate Problem.

The creation in France of the new Ministry of Hygiene has had as its first result the establishment of a Government Commission, the object of which is to combat depopulation, increase the birth-rate, develop child welfare, and protect large families. Hitherto the question of the future of the race has been left to the care of unofficial leagues, societies, and congresses; the establishment of a permanent Government organisation is, however, in the view of M. Breton, the new Minister, a necessity.

The manner in which the Commission will carry out its task will be, apparently, first by examining from the point of view of the effect upon the birth-rate not only of all projects of legislation to be framed in the future, but those already in force, and reporting upon them; secondly, to discover what is being done by private initiative to encourage large families; thirdly, by instituting in every department a local birth-rate commission under the presidency of the Prefect of the Department, three of the members of which must be fathers of large families. These departmental commissions will act as the agents on the spot for the main Commission, which will co-ordinate the information thus received and act upon it. According to a writer in the *Petit Journal*, who gives the above outline of the Commission's activities after a conversation with M. Breton, the duties of these departmental commissions will also include the carrying on of propaganda among the local population.

But the difficulty of those who, whether in France, England, or elsewhere preach large families, is that it is so largely an economic question. The efficient up-bringing of a family of three is of more value to a State than the inefficient dragging up of five. Even so, there is plenty of ground to cover in persuading the selfish to undertake the privileges and responsibilities of parenthood.

